# A Prospective Clinical Evaluation of the Retention of Glass Ionomer Cement Versus Resin-Based Pit and Fissure Sealants Among School Children

**DOI:** 10.7759/cureus.102037

**Published:** 2026-01-21

**Authors:** Debadrita Ghosh, Sunita Shivanand, Piyushi Tiwari, Deepak Kolte, Shamurailatpam Priyadarshini, Dilip Katakam, Rahul Tiwari, Seema Gupta

**Affiliations:** 1 Department of Pedodontics and Preventive Dentistry, Family Dental Clinic, Kolkata, IND; 2 Department of Conservative Dentistry and Endodontics, KLE Vishwanath Katti Institute of Dental Sciences and Hospital, Belagavi, IND; 3 Department of Conservative Dentistry and Endodontics, RKDF Dental College and Research Centre, Bhopal, IND; 4 Department of Oral and Maxillofacial Surgery, Bharati Vidyapeeth (Deemed to be University) Dental College and Hospital, Mumbai, IND; 5 Department of Conservative Dentistry and Endodontics, The Dental College, Regional Institute of Medical Sciences, Imphal, IND; 6 Department of Conservative Dentistry and Endodontics, ESIC Dental College and Hospital, Kalaburagi, IND; 7 Department of Oral and Maxillofacial Surgery, RKDF Dental College and Research Centre, Bhopal, IND; 8 Department of Orthodontics, Kothiwal Dental College and Research Centre, Moradabad, IND

**Keywords:** children, dental caries, glass ionomer cement, pit and fissure sealants, resins

## Abstract

Introduction

Dental caries are commonly observed in schoolchildren, particularly on the complex pit and fissure surfaces of permanent molars, which readily trap plaque and debris, making sealants a critical preventive intervention. This prospective comparative clinical study sought to assess the retention rates and clinical performance of glass ionomer cement (GIC) and resin-based sealants in a school-based setting among children aged 7-10 years.

Materials and methods

This study was carried out in government primary schools in Bhopal, India, from June to December 2024. Eighty children with at least one caries-free permanent first molar were non-randomly assigned to two groups (n = 40 per group). Group 1 received a high-viscosity glass ionomer cement (GIC) sealant (Fuji IX GP; GC Corp., Tokyo, Japan), applied using the press-finger technique following polyacrylic acid conditioning. Group 2 received a light-cured, fluoride-releasing resin-based sealant (Clinpro; 3M ESPE, 3M Company, St. Paul, MN), applied after phosphoric acid etching. Sealants were placed by a single operator using a portable device. Retention was evaluated at five months using a custom three-point scoring system (complete, partial, or complete loss), with high intra- and inter-examiner reliability (kappa ≥0.85). Data were analyzed using Chi-square, Mann-Whitney U, and Kaplan-Meier survival tests (p<0.05).

Results

Resin-based sealants showed significantly higher retention, with 38% complete retention compared with 26% for GIC, and lower failure rates (13% versus 24%; p = 0.031). Survival analysis demonstrated greater longevity of resin-based materials, with fissure sites performing better than pits (p = 0.001). Twice-daily toothbrushing was associated with better sealant retention (p = 0.021). No significant differences were found with respect to age, sex, or diet.

Conclusions

Resin-based sealants demonstrated superior short-term retention and durability compared with GIC in schoolchildren. The choice of material should take into account the clinical setting and patient-related factors to optimize caries prevention.

## Introduction

Dental caries remains a major public health concern among children worldwide, particularly in school-age populations, where permanent molars are especially vulnerable. The occlusal surfaces of these teeth, which are characterized by deep pits and fissures, often trap food debris and bacterial plaque, making them susceptible to decay even with regular brushing [1,2]. Pit and fissure caries constitute a substantial proportion, often exceeding 90%, of total caries in children’s permanent teeth, contributing markedly to the overall disease burden [3]. Preventive strategies are therefore critical, particularly in resource-limited settings, where access to restorative care is limited. Pit and fissure sealants are a minimally invasive, evidence-based intervention that forms a protective barrier over susceptible surfaces, significantly reducing the risk of caries development [2]. Systematic reviews have shown that sealants effectively prevent occlusal caries in high-risk children when properly applied and maintained [4,5].

Two primary sealant materials are widely used: resin-based sealants and glass ionomer cement (GIC) [6]. Resin-based sealants provide excellent retention due to a strong mechanical bond created after acid etching, along with favorable aesthetics and durability. However, their placement is highly technique sensitive, requiring strict moisture control, which can be challenging in uncooperative children or in community-based school programs [7]. In contrast, GIC sealants are more tolerant of moisture, simpler to apply under field conditions, and release fluoride over time, promoting remineralization and providing cariostatic benefits even when partial retention is lost [8]. Although GIC sealants have lower long-term retention rates compared with resin-based materials [8], they often produce comparable caries-preventive outcomes, making them particularly well suited for school-based programs in underserved settings.

Given the respective strengths and limitations of each material, direct comparisons in real-world school settings are necessary to inform clinical decision-making. Accordingly, this study sought to comparatively evaluate the effectiveness of high-viscosity glass ionomer cement (HVGIC) and resin-based sealants in preventing pit and fissure caries in schoolchildren. The objectives were to determine the retention rates of HVGIC and resin-based sealants over a specified follow-up period, compare the incidence of pit and fissure caries in teeth sealed with each material, and assess clinical performance, including marginal integrity and the occurrence of any adverse effects, in a school-based population.

## Materials and methods

Study design

This prospective, comparative clinical study was conducted in the Department of Conservative Dentistry and Endodontics, RKDF Dental College and Research Centre, Bhopal, India. It was designed as a non-randomized study, with participants allocated to one of the two sealant groups based on their sequential order of enrolment and parental preference, ensuring ethical considerations while maintaining prospective follow-up. No split-mouth design was employed to avoid potential cross-contamination or bias from intraindividual comparisons.

Study setting and duration

The study was conducted in selected government primary schools in an urban area with moderate caries risk. Participant recruitment and sealant application occurred over one month, followed by a six-month follow-up evaluation. The total study duration was approximately seven months (June 2024 to December 2024).

Study population and sample size

Schoolchildren aged 7 to 10 years with at least one fully erupted, caries-free permanent first molar, confirmed clinically and radiographically when necessary, were eligible for inclusion in the study. Exclusion criteria included teeth with enamel hypoplasia, existing restorations, visible cavitation, developmental defects, or a prior history of sealant placement. Informed consent was obtained from parents or guardians, and assent was obtained from the children.

The sample size was calculated using G*Power software (version 3.1.9.2; Heinrich Heine University, Düsseldorf, Germany). Based on a reported success rate of 66% for resin-based sealants in a previous study [9], a minimum of 40 samples per group was required to achieve 80% statistical power with a two-sided alpha error of 5%.

Materials used

Group 1 (n = 40) used a GIC sealant (Fuji IX GP, GC Corp., Tokyo, Japan), which is a high-viscosity conventional GIC used as a pit and fissure sealant. Group 2 (n = 40) used a resin-based sealant (Clinpro sealant, 3M ESPE, St. Paul, MN), which is a light-cured, filled resin-based pit and fissure sealant containing fluoride. Although the two materials differ in their primary manufacturer-indicated classifications, both were selected for the same preventive purpose of pit and fissure sealing. HVGIC, though commonly categorized as a restorative material, is well documented in the literature for use as a fissure sealant, particularly in atraumatic restorative treatment and school-based preventive programs due to its chemical adhesion to enamel, fluoride release, and tolerance to moisture. The resin-based material represents the conventional sealant routinely used in clinical practice. Each material was applied strictly according to manufacturer-recommended protocols, and differences in application technique reflect inherent material properties rather than methodological inconsistency. The comparison was therefore intentional, aiming to assess their relative clinical performance and retention under real-world school settings, where such material choices are commonly encountered.

Sealant application procedure

All procedures were performed by a single, calibrated operator in a school setting using portable dental equipment. The teeth were isolated with cotton rolls, cleaned with a toothbrush and pumice slurry (non-fluoridated), and dried with compressed air or gauze. For Group 1, the occlusal surface was conditioned with 10% polyacrylic acid for 10 seconds (where applicable), rinsed, and lightly dried without desiccation. The material was mixed according to the manufacturer’s instructions, applied using the press-finger technique to ensure adaptation into the fissures, and protected with a thin layer of petroleum jelly immediately after placement. For Group 2, the occlusal surface was etched with 37% phosphoric acid gel for 15-20 seconds, rinsed thoroughly for 10 seconds, and dried until a frosty white appearance was achieved. The sealant was applied using the manufacturer’s syringe tip, gently spread into pits and fissures, and light-cured for 20 seconds using a light-emitting diode (LED) curing unit. Post-application instructions were provided to avoid eating hard foods for 24 hours.

Follow-up and clinical evaluation

All participants were recalled at one, three, and six months (±1 week) for clinical assessment of sealant retention. Evaluation was performed under good illumination using a mouth mirror and explorer by the same calibrated operator. Operator blinding was not feasible in this study. The participants, parents, and school staff remained unaware of which sealant was applied to each child’s teeth.

Retention scoring criteria

Sealant retention was clinically assessed at one-, three-, and six-month follow-ups using a three-point scoring system developed specifically for this study. Score 1 indicated complete retention (sealant fully present, covering all pits and fissures with no detectable loss of material). Score 2 indicated partial retention (sealant partially present with some material loss, but still covering at least 50% of the pits and fissures or remaining adherent in the deeper grooves). Score 3 indicated a complete loss (no detectable sealant material remaining on the occlusal surface). This custom scoring system was designed to provide a straightforward, reproducible clinical evaluation tailored to the objectives of the present study, without reliance on previously published external criteria. Marginal integrity and surface wear were qualitatively observed but not scored independently, as the primary outcome focused on retention.

Before the commencement of the study, the outcome assessor underwent a rigorous calibration process to ensure the reliable and reproducible application of the custom 3-point retention scoring system. Calibration involved the examination of 30 permanent molars with varying degrees of sealant retention (complete, partial, and complete loss), selected from clinical photographs, and in vivo teeth not included in the main study sample. The assessor scored each tooth twice, with a two-week interval between the first and second assessments to evaluate intra-examiner reliability. Additionally, the scores were independently compared with those of an experienced pediatric dentist serving as a reference examiner (gold standard) to assess inter-examiner reliability. The process was repeated until a weighted Kappa value of ≥0.85 was achieved for both intra- and inter-examiner agreement, indicating excellent reproducibility and consistency in the interpretation of the criteria. This high level of reliability confirmed that the custom scoring system could be applied uniformly throughout the study, thereby minimizing subjective variations in clinical evaluations. Data on age, sex, tooth number, sealant type, and retention scores were recorded. 

Ethical considerations

The study protocol was approved by the Institutional Ethics Committee of RKDF Dental College and Research Centre (RKDF/DC/2024/S12). Participation was voluntary, and standard infection control protocols were followed throughout the study.

Statistical analysis

Data were analyzed using SPSS Statistics software (IBM Corp., Armonk, NY). A chi-square test was used to measure the differences between the parameters for retention scoring (ordinal data). The Mann-Whitney U test was used to compare the mean retention scores at different time periods. A survival analysis of the different sealant materials was performed using the Kaplan-Meier method and the Cox proportional hazard model. The significance level was set at p<0.05.

## Results

The analysis of the association with sealant retention scores is presented in Table 1. Among the evaluated parameters, three demonstrated statistically significant relationships. The type of material used showed a significant association (p = 0.031), with GIC exhibiting a higher proportion of the lowest retention scores. A strong association was found with the type of dental filling, where pit seals were linked to poorer retention scores compared to fissure seals (p = 0.001). Furthermore, brushing frequency was significant (p = 0.021), indicating better retention with twice-daily brushing. No significant associations were found between the patients’ sex, age group, or diet (all p>0.05). These results suggest that the choice of restorative material, the anatomical site being sealed, and the patient's oral hygiene practices are key factors influencing sealant retention in this cohort.

**Table 1:** Association between sealant retention scores and demographic, clinical, and behavioral parameters at the five-month follow-up ^*^P<0.05 considered statistically significant Sealant retention scores: 1 = complete retention; 2 = partial retention; 3 = complete loss; χ² test used for association HVGIC: high-viscosity glass ionomer cement

Parameters	Category	Sealant retention score			Chi stats (χ²)	P-value
		Score 1, n (%)	Score 2, n (%)	Score 3, n (%)		
Sex	Male	16 (20%)	12 (15%)	14 (18%)	0.66	0.719
	Female	15 (19%)	8 (10%)	15 (19%)		
Age group, years	7	7 (9%)	4 (5%)	9 (11%)	2.91	0.819
	8	7 (9%)	4 (5%)	9 (11%)		
	9	8 (10%)	6 (8%)	6 (8%)		
	10	9 (11%)	6 (8%)	5 (6%)		
Sealant material	HVGIC	10 (13%)	11 (14%)	19 (24%)	6.89	0.031^*^
	Resin-based	21 (26%)	9 (11%)	10 (36%)		
Sealant location	Pit	7 (9%)	2 (3%)	18 (23%)	17.17	0.001^*^
	Fissure	24 (30%	18 (23%)	11 (14%)		
Diet	Vegetarian	19 (24%)	12 (15%)	20 (25%)	0.54	0.761
	Non-vegetarian	12 (15%)	8 (10%)	9 (11%)		
Brushing frequency/day	Once	17 (22%)	14 (18%)	25 (31%)	7.02	0.021^*^
	Twice	14 (18%)	6 (8%)	4 (5%)		

The analysis of sealant success rates revealed a statistically significant difference between the materials. The resin-based sealant demonstrated a higher success rate of 38% compared with 26% for HVGIC. Correspondingly, the failure rate for HVGIC was notably higher at 24% compared to 13% for the resin-based group. This suggests that resin-based sealants provide superior clinical performance and retention in this study population. The findings indicate that material choice is a critical factor, with resin-based formulations being a more reliable option for achieving long-term sealant success under the given conditions (Table 2).

**Table 2:** Comparison of sealant success and failure rates between materials ^*^P<0.05 considered statistically significant Success is defined as complete or partial retention. Failure is defined as the complete loss of sealant. χ² test applied HVGIC: high-viscosity glass ionomer cement

Outcome	HVGIC	Resin-based	χ²	P-value
Success	21 (26%)	30 (38%)	4.31	0.036^*^
Failure	19 (24%)	10 (13%)		

The longitudinal comparison of retention scores revealed a consistent and statistically significant advantage of resin-based sealants at every follow-up interval. At one, three, and six months, the resin-based group demonstrated superior median retention scores (lower scores indicated better retention) with tighter interquartile ranges (IQRs) than the HVGIC group. This trend indicates not only better initial performance but also more predictable and stable retention over time for resin-based materials (Table 3).

**Table 3:** Comparison of retention score at different time points with Mann-Whitney U test ^*^P<0.05 considered statistically significant Lower median scores indicate better retention. Mann-Whitney U test used for comparison HVGIC: high-viscosity glass ionomer cement; IQR: interquartile range

Follow-up period	HVGIC sealant		Resin-based sealant		U value	P-value
	Median	IQR	Median	IQR		
1 month	2	2	1	1	549.0	0.016^*^
3 months	3	2.5	2	1.2	435.5	0.046^*^
6 months	2	2	1	1	478.0	0.025^*^

**Figure 1:**
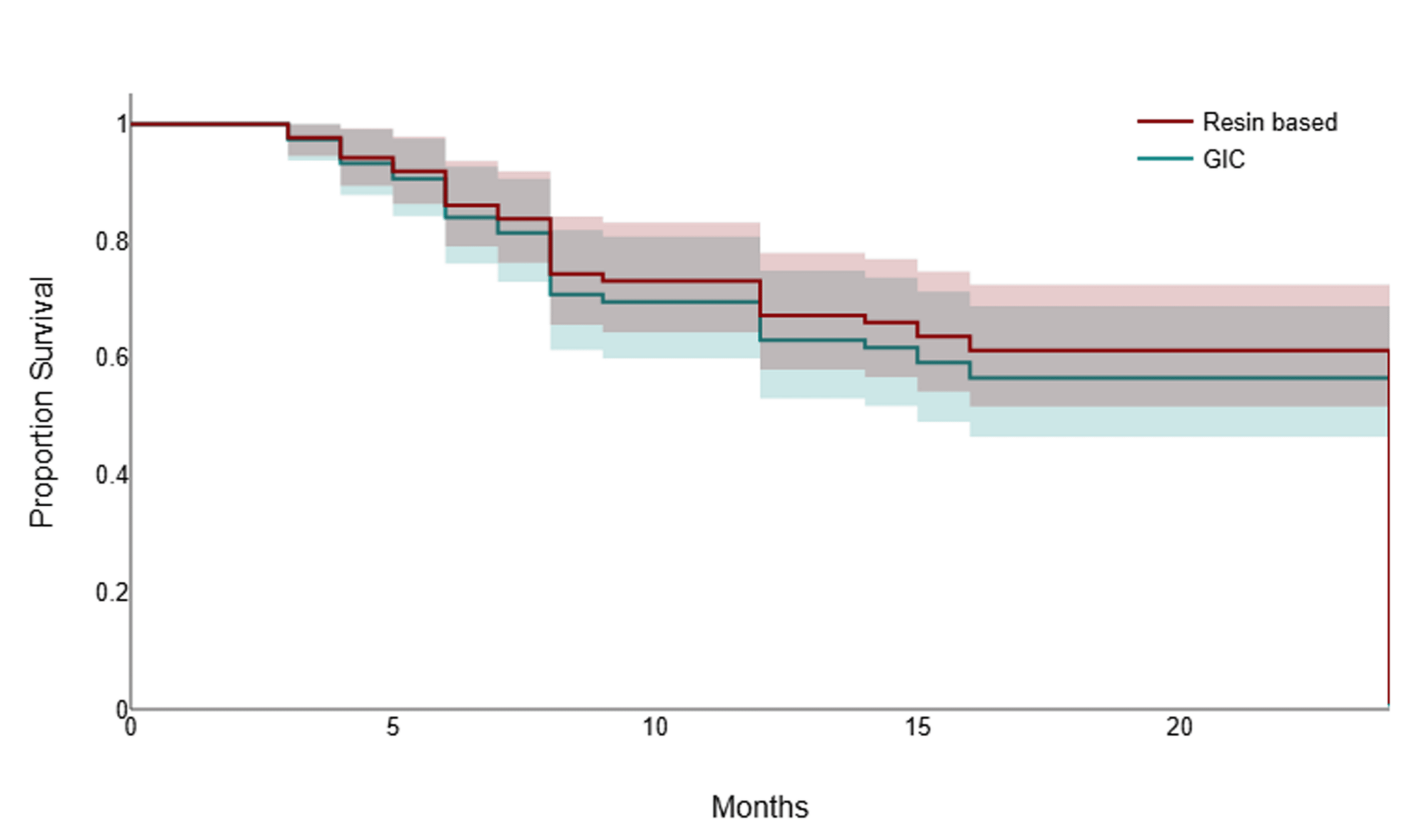
Kaplan-Meier survival analysis comparing sealant retention between pit and fissure locations GIC: glass ionomer cement

The survival probability of sealants placed in fissures was consistently and markedly higher than that of sealants placed in pits at all time points. The curves began to diverge early, indicating that pits were associated with a higher rate of early sealant failure. By the end of the 24-week observation window, the estimated proportion of surviving sealants remained substantially greater in the fissure group. This result strongly suggests that the anatomical site, specifically sealants placed in pits, is a critical risk factor for retention loss, with pit cavities showing significantly poorer survival rates than fissure cavities (Figure 1).

**Figure 2:**
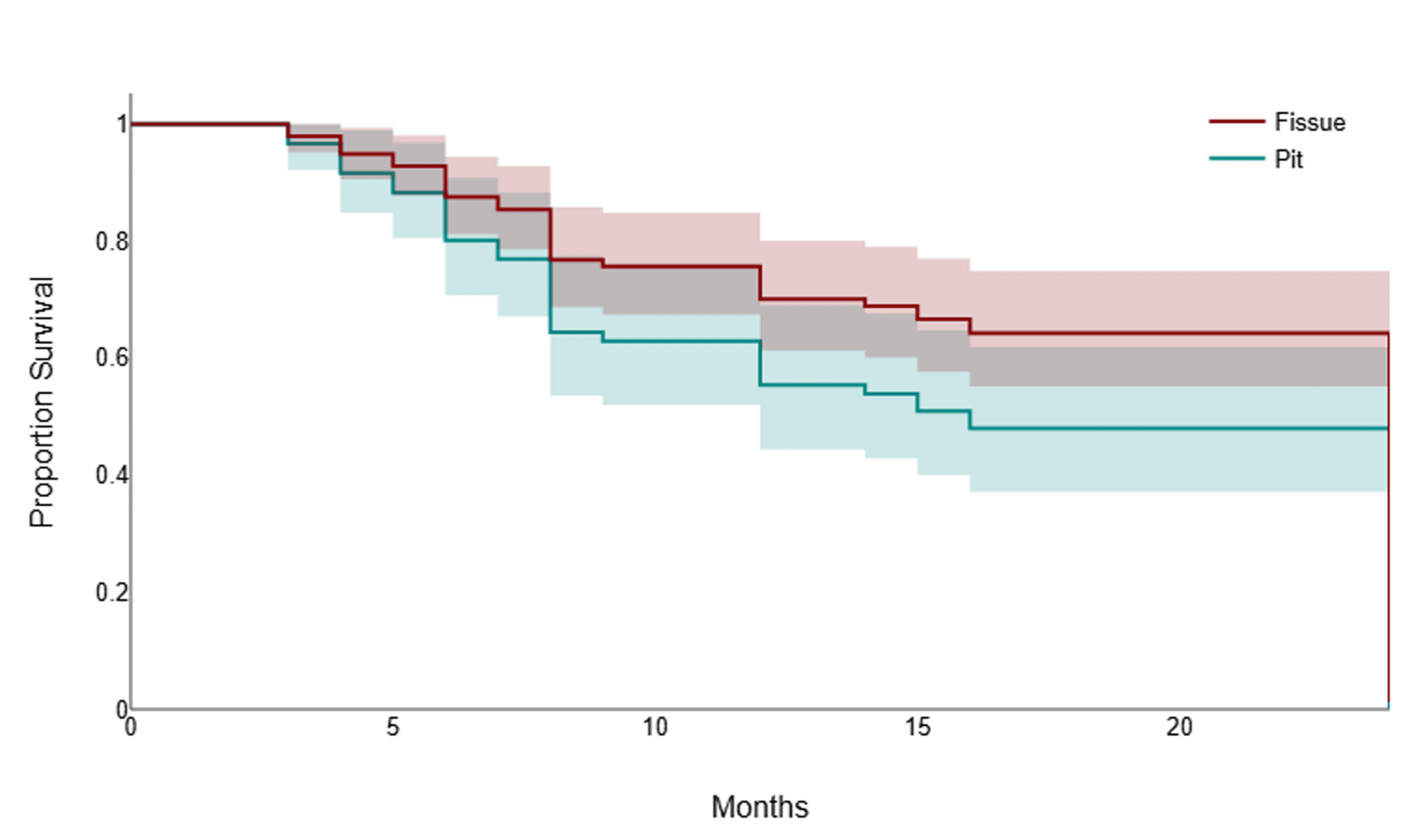
Kaplan-Meier survival analysis comparing retention of HVGIC and resin-based sealants HVGIC: high viscosity glass ionomer cement

Kaplan-Meier survival analysis showed that the type of sealant material significantly influenced retention over the 24-week follow-up period. The survival curve for resin-based sealants demonstrated consistently superior retention, maintaining a higher probability of survival compared to that of HVGIC at every observed time point. The curves diverged early and continued to separate over time, indicating a higher and more rapid rate of failure in the HVGIC group. By the end of the six-month observation period, the estimated proportion of fully retained sealants was substantially greater for resin-based materials. This result strongly suggests that resin-based sealants provide clinically superior and more durable retention in the short to medium term (Figure 2).

## Discussion

The findings of the present investigation revealed that resin-based sealants exhibited superior retention compared to HVGIC sealants in preventing pit and fissure caries in school-aged children. Over six months, resin-based materials achieved a 38% success rate in complete retention, contrasting with 26% for HVGIC, whereas failure rates stood at 13% and 24%, respectively. The differences were statistically significant. Additionally, sealants applied to fissures demonstrated better survival probabilities than those applied to pits, and twice-daily brushing was associated with improved retention outcomes. No notable links emerged between retention and factors such as age, sex, and diet.

These observations are consistent with those of multiple clinical trials and systematic reviews. For instance, a systematic analysis of 13 randomized controlled trials (RCTs) indicated that resin-based sealants consistently outperformed GIC in retention rates, although both materials showed similar efficacy in caries prevention [10]. In a 12-month RCT involving children aged 7-10 years, resin sealants displayed significantly higher retention, with GIC experiencing more total loss; however, no new caries developed in either group [11]. A long-term 10-year follow-up study reinforced this, reporting a higher survival rate for resin-based sealants at one year compared to GIC, as shown in Kaplan-Meier survival curves [9]. These patterns suggest that mechanical bonding of resin via acid etching provides greater durability, particularly on occlusal surfaces under masticatory stress.

The disparity in retention between pits and fissures may stem from their anatomical variations. Pits, which are often deeper and narrower, pose challenges for material penetration and adaptation, leading to higher early failure rates. Fissure morphology influences outcomes, with U- or V-shaped grooves allowing better sealant flow than I-shaped grooves, which restrict penetration and increase the risk of microleakage. These findings underscore the need for careful site assessment during application to optimize the longevity of the restoration [12,13]. Similar findings were reported by Garg et al. [14], who found that GIC sealants provided better penetration than filled and unfilled resin-based sealants.

The incorporation of filler particles into resin-based sealants modifies the viscosity and diminishes the sealant's capacity to infiltrate fissures and microporosities within the etched enamel. No pit and fissure sealant can effectively infiltrate the depths of elongated and constricted fissures and deep pits; thus, certain clinicians may hypothesize the presence of microorganisms in inadequately filled areas or presume that the sealant is frequently applied atop an incipient carious lesion. Nonetheless, there is evidence indicating that bacteria cannot sustain viability and that the progression of carious lesions ceases when a sealant is applied over an incipient lesion [14].

Our study also reported a positive association between oral hygiene and twice-daily brushing. However, a previous study found no association between the type of toothbrushing and sealant retention [15]. Improved plaque removal likely reduces bacterial challenges and abrasive wear, preserving the sealant-tooth interface. A review of preventive care emphasized that regular brushing bolsters sealant effectiveness by minimizing debris accumulation in sealed areas [7]. Nonetheless, promoting frequent brushing remains vital for synergistic prevention of caries.

Clinically, these results support the use of resin-based sealants in settings where moisture control is feasible, given their superior retention and stability over time. In school-based programs, where absolute isolation may be challenging, GIC's moisture tolerance and fluoride release offer advantages, potentially compensating for lower retention through remineralization. Despite inferior retention, GIC's caries-preventive efficacy often matches that of resin. This supports tailored material selection: resin for cooperative patients in controlled environments and GIC for field applications in underserved communities. Integrating sealants with oral hygiene education could amplify the benefits and reduce the incidence of caries. Survival analyses further imply that early interventions in fissures yield better long-term results, thereby informing targeted prevention strategies.

Limitations

The limitations of this study should be acknowledged. The six-month follow-up period is relatively short; longer-term studies, such as the 10-year evaluation cited, demonstrate progressive differences in retention, highlighting the need for extended monitoring. The non-randomized design, based on sequential enrollment and parental preference, introduces potential selection bias, although ethical considerations supported this approach. Operator blinding was impractical, posing a risk of assessment bias despite calibration achieving high Kappa values. Although the sample size was powered for primary outcomes, it limits the generalizability to diverse populations or higher caries-risk settings. School-based implementation using portable equipment may not fully reflect clinical office conditions, and custom retention scoring, though reliable, has not been externally validated against standardized criteria. Adverse effects were only minimally addressed, and caries incidence was not directly measured, focusing on retention as a proxy.

Future research directions

Future research should employ randomized, blinded designs with multi-year follow-up to validate these findings. Exploring hybrid materials or adjuncts, such as bonding agents, could improve retention in challenging anatomies. Overall, this study provides evidence supporting resin-based sealants for durability while emphasizing GIC's role in accessible prevention, ultimately informing cost-effective strategies to reduce childhood caries.

## Conclusions

This prospective clinical study demonstrated that resin-based pit and fissure sealants exhibited significantly superior retention compared to HVGIC sealants over a six-month period in school children, with higher complete retention rates and better survival probabilities. Factors such as fissure anatomy and frequent brushing further enhance the longevity of sealants. However, HVGIC's moisture tolerance and fluoride release make it valuable in resource-limited school settings, often achieving comparable caries prevention despite lower retention. Tailored selection of sealants, combined with oral hygiene promotion, can effectively reduce the burden of occlusal caries in pediatric populations.
